# Circular RNAs: Biogenesis, Mechanism, and Function in Human Cancers

**DOI:** 10.3390/ijms20163926

**Published:** 2019-08-13

**Authors:** Xing Zhao, Yujie Cai, Jianzhen Xu

**Affiliations:** 1 Computational Systems Biology Lab, Department of Bioinformatics, Shantou University Medical College (SUMC), No. 22, Xinling Road, Shantou 515041, China; 2 Department of Pathology and Medical Biology, University of Groningen, University Medical Center Groningen, 9700 RB Groningen, The Netherlands

**Keywords:** circRNAs, carcinogenesis, biogenesis, RNA-binding protein, flanking introns, genomic alternation

## Abstract

CircRNAs are a class of noncoding RNA species with a circular configuration that is formed by either typical spliceosome-mediated or lariat-type splicing. The expression of circRNAs is usually abnormal in many cancers. Several circRNAs have been demonstrated to play important roles in carcinogenesis. In this review, we will first provide an introduction of circRNAs biogenesis, especially the regulation of circRNA by RNA-binding proteins, then we will focus on the recent findings of circRNA molecular mechanisms and functions in cancer development. Finally, some open questions are also discussed.

## 1. Introduction

Discovered more than three decades ago [1], circRNAs have attracted extensive attention in recent years [2,3,4]. As a member of the noncoding RNA family, circRNAs display a unique covalently closed circular form, which distinguishes it from its other noncoding RNA cousins such as microRNA and lncRNA. High-throughput RNA-seq studies have detected a large number of circRNAs with different lengths and types. Initial analyses of the sequencing data indicate they are specifically expressed during tissue/developmental stages and conserved between mice and humans [3,4]. Emerging evidence has demonstrated that some circRNAs have various biological functions and play potentially important roles in multiple diseases such as cancers [5,6,7].

Here, we summarize the expanding findings on circRNA and provide an up-to-date account of their biogenesis, regulatory mechanisms, and cellular functions in carcinogenesis.

## 2. The Biogenesis of circRNAs in Cancers

In eukaryotes, linear mRNA is formed by processing exons of pre-mRNAs through alternative splicing. Unlike linear RNAs, circRNAs were originally thought to be aberrant RNA splicing products [8]. Because of their covalent closed-loop structure, circRNAs are protected from degradation by RNases, so they are more stable than linear RNAs [3]. When analyzing 17 different cancer cohorts from the MiOncoCirc compendium, Vo et al. characterized circRNAs across >2000 cancer samples and observed a general decrease in total circRNA abundance compared to the adjacent normal tissues [9].

**Figure 1:**
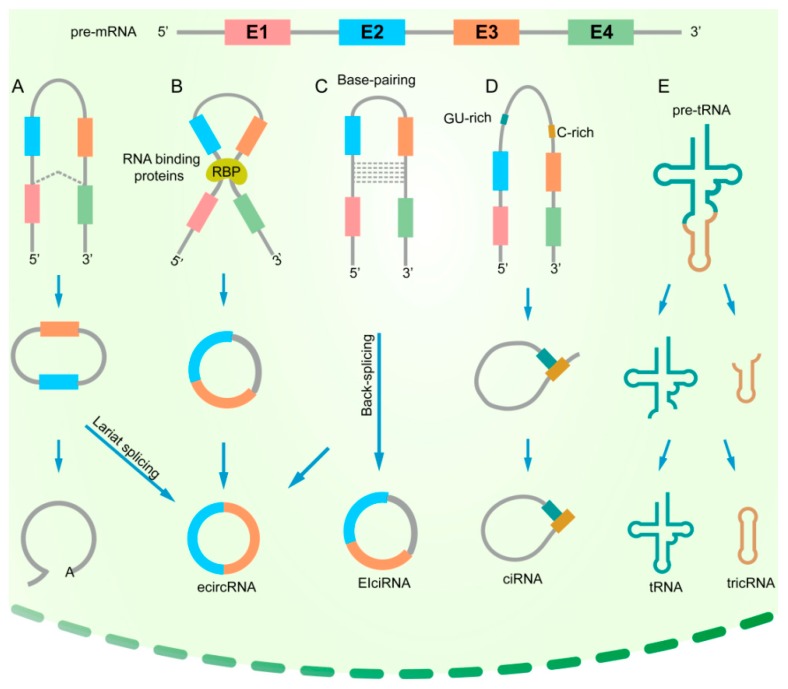
Biogenesis of circRNAs. (**A**) Lariat-driven circularization. When a pre-mRNA is spliced, the 3′ hydroxyl of the upstream exon interacts with the 5′ phosphate of the downstream exon to form a covalent linkage, producing a lariat that contains exons and introns. The 2′ hydroxyl of the 5′ intron reacts with the 5′ phosphate of the 3′-intron, followed by an interaction between the 3′ hydroxyl of the 3′ exon and the 5′ phosphate of the 5′ exon, through which an ecircRNA is formed. (**B**) RNA-binding protein (RBP)-driven circularization. RBPs can promote the interaction of the downstream intron and upstream intron, causing the formation of an ecircRNA. (**C**) Base-pairing-driven circularization. The downstream introns and upstream introns are paired based on inverse-repeating or complementary sequences. The introns are removed or retained to form ecircRNA or EIciRNA. (**D**) Biosynthesis of ciRNA. Formation of ciRNAs mainly depends on a 7-nt GU-rich element and an 11-nt C-rich element to escape debranching and exonucleolytic degradation. (**E**) Formation of tricRNA. tRNA splicing enzymes divide pre-tRNA into two parts: tricRNAs are generated by a 3′–5′ phosphodiester bond, and the other part generates tRNAs.

With the optimization of next-generation sequencing technology, several circRNA subtypes have been discovered in recent years. There are four main subtypes of circRNAs: exonic circRNAs (ecircRNAs), which are mainly derived from single or several exons; on the other side, circular intronic RNAs (ciRNAs) contain only introns; exonic-intronic circRNAs (EIciRNAs), which consist of both introns and exons; and tRNA intronic circRNAs (tricRNAs) are formed by splicing pre-tRNA intron (Figure 1). Currently, most of the identified circRNAs are exonic circRNAs.

The formation mechanisms of different types of circRNAs are also diversely regulated. Generation of exonic circRNAs can be achieved by both lariat-driven circularization and back splicing. When the upstream splice acceptor and downstream donor are close to forming a lariat containing the exons, the introns in the lariat are removed, and the exons are joined by a 5′–3′ phosphodiester bond (Figure 1A) [10]. Interactions between RNA-binding proteins (RBPs) form a ‘bridge’ within the upstream and downstream introns, followed by back splicing to form ecircRNAs (Figure 1B) [11,12]. During base-pairing-driven circularization, the downstream splicing donor is connected to the upstream splicing receptor depending on ALU complementary sequences. In the process of circRNA formation, the introns are removed or retained to form ecircRNA or EIciRNA(Figure 1C) [3]. The biosynthesis of ciRNAs mainly depends on a 7-nt GU-rich element and an 11-nt C-rich element to escape debranching and exonucleolytic degradation (Figure 1D) [13]. Unlike ecircRNAs, which are mainly located in the cytoplasm, ciRNAs and EIciRNAs are mainly distributed in the nucleus and play a crucial role in regulating parental gene transcription. A special intronic circRNA, tricRNA, has been found in *Archaea* and *Drosophila* [14,15,16]. The formation of tricRNA needs tRNA splicing enzymes to divide pre-tRNA into two parts: tricRNAs are generated by a 3′–5′ phosphodiester bond, and the other part generates tRNAs (Figure 1E) [15]. It is generally accepted that both the binding of RNA proteins and the presence of specific and repetitive sequences in the introns surrounding the circularizing exons determine the production of circRNAs.

### 2.1. Introns Flank circRNAs 

Previously, analysis of the surrounding introns of circRNAs in human fibroblasts has indicated they are usually longer and harbor complementary ALU repeats [3]. A recent analysis of circRNAs in over 2000 cancer samples confirmed this finding [9]. These repetitive sequences are thought to facilitate circular RNA formation [17]. Besides, Ivanov et al. found that the reverse complementary sequences are conserved and highly enriched within introns that bracket circRNAs. They also successfully developed a computational system to identify novel circRNAs based on scoring the presence of reverse complementary sequences in human introns [18]. Furthermore, introns upstream or downstream circularized exons, are preferentially harbor more RNA editing events, which proved to affect the formation of circRNA [18,19].

### 2.2. Regulation of circRNA Biogenesis by RNA-Binding Proteins (RBPs)

RNA-binding proteins, which usually contain RNA-binding motifs, play a central role in transcriptional regulation of genes [20]. Because of the development of high-throughput methods, such as RNA sequencing and mass spectrometry, about ~1500 RBPs have been identified from the human genome until now [21,22]. Bioinformatics analyses of the alteration spectrum across thousands of human cancers samples identified ~9% of cancer-related RBPs, highlighting its potential roles in tumorigenesis [23]. RBPs can shorten the distance between the donor site and the receptor site by binding to the introns on the flank regions, thus promoting circularization of the exons in circRNA biogenesis. 

*QKI*, KH domains containing RNA binding proteins, is the first identified RBP involved in circRNA formation during the epithelial to mesenchymal transition. It enhances circRNA formation by binding to its consensus target motif, single-stranded RNA (ssRNA), in introns that flank the circRNA-forming exons. Consequently, insertion of synthetic *QKI* binding sites into introns was sufficient to produce circRNAs [12]. Based on genome-wide siRNA screening and circRNA expression reporter assays, Li et al. identified that the immune factors nuclear factor 90 and its 110 isoform (*NF90/NF110*) could couple circRNA biogenesis and function during viral infection. They found the nuclear export of *NF90/NF110* to the cytoplasm correlated with decreased circRNA expression, while NF90/NF110 bound to viral mRNAs to stimulate an antiviral immune response [24]. Epithelial splicing regulatory protein 1 (*ESRP1*) is an essential splicing factor during pluripotency [25]. A recent report found an elegant regulating network involved in circRNA and RNA-binding protein in human embryonic stem cells (hESCs). In this scenario, *NANOG* and *OCT4* regulate *ESRP1* expression in hESCs, which leads to the promotion of *circBIRC6* generation. Consequently, *circBIRC6* works as a ‘molecular reservoir’ of *miR-34a* and *miR-145*, collectively contributing to pluripotency maintenance [26]. 

Different RBPs may play different, or even opposite, roles in the back-splicing process. For example, all the above three RBPs can promote the production of circRNAs, whereas the RNA-editing enzyme (that edits adenosine to inosine) acts on RNA enzyme 1 (*ADAR1*) to inhibit circRNA formation [18]. *ADAR1* binds double-stranded RNA to mediate adenosine-to-inosine (A-to-I) RNA editing [27]. Large scale analyses of multiple cancer samples from The Cancer Genome Atlas (TCGA) indicate RNA editing events in tumor samples correlate best with the *ADAR1* expression level globally. Approximately 3.5% of detected RNA editing sites are associated with potential clinical relevance, many of which are in noncoding regions [19]. This is consistent with a report that A-to-I editing is also enriched with the base-paired or proximal regions of circularized exons [18]. A mechanistic model suggested RNA editing sites may disrupt the RNA–RNA interactions of introns to form larger structures [28]. This is supported by the evidence that *ADAR1* depletion can up-regulate the formation of circRNAs [18]. The known instances of RBPs have been summarized in Table 1.

### 2.3. Impacts of Genomic Alterations on the Formation of circRNAs

Oncogenic gene fusion is commonly found in cancer samples and plays a role in carcinogenesis. Guarnerio et al. hypothesized chromosome rearrangement will result in the juxtaposition at a close enough proximity to favor new events of back splicing, which would promote the generation of aberrant circRNAs [29]. To verify this hypothesis, they examined the most recurrent translocation fusion genes, such as *PML-RARa* and *MLL/AF9* fusion genes, in leukemia. Circular forms of fusion circRNAs (f-circRNA) are indeed produced from transcribed exons of distinct genes and affected by the translocations. Furthermore, they also found circular *EWSR1/FLI1* fusion in SK-NEP-1 sarcoma cells and *EML4/ALK1* in H3122 lung cancer cells. However, a recent search of MiOncoCirc data, which included 17 cancer sequencing datasets, did not detect any f-circRNAs resulting from chromosomal translocations and deletions. Instead, they discovered read-through circRNA (rt-circRNA), a novel class of circular transcripts that span exons originating from two adjacent genes on the same strand. They found some of these rt-circRNA reads were commonly found across different cancer types, and they were even detected in normal tissues or in samples with a normal copy number of the parent genes, which indicated the prevalence of rt-circRNAs in cancer samples [9].

## 3. Functional Mechanisms of circRNAs

**Figure 2:**
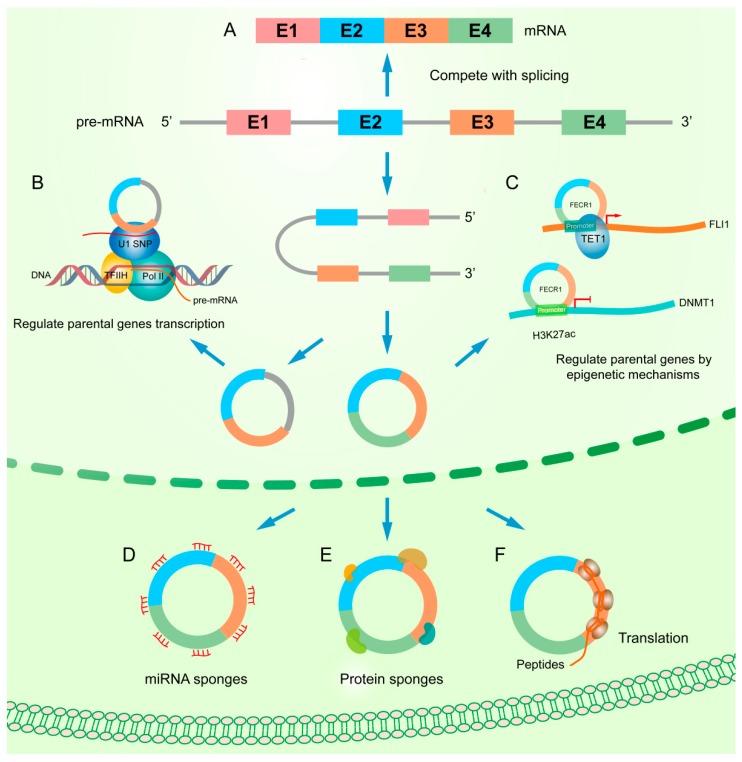
The molecular mechanism of circRNAs (**A**) Competition with splicing. The formation of circRNA competes with the splicing of linear RNA. (**B**) Regulation of parental gene transcription. ciRNAs and EIciRNAs, with retained introns, can bind to U1 snRNP through RNA–RNA interactions and further interact with the Pol II transcription complex to enhance parental gene expression. (**C**) Circular RNA *FECR1* from *FLI1* gene interacts with *FLI1* promoter, recruits *TET1* demethylase, and induces extensive DNA demethylation in the CpG islands. In addition, *FECR1* inhibits *DNMT1*, a critical enzyme that maintains DNA demethylation during DNA replication, by binding to its promoter region rich in *H3K27ac*. (**D**) miRNA sponges. CircRNA affects the expression of miRNA and downstream target genes by adsorbing miRNAs. (**E**) Protein sponges. CircRNA regulates target proteins by binding to them. (**F**) Translation. CircRNAs can be translated into peptides or proteins.

The functional mechanism of circRNAs are diverse in human cancer including acting as miRNA sponges, interacting with protein, regulating gene splicing or transcription, translating proteins, or peptide and epigenetic regulation (Figure 2). According to the targets of circRNAs, they can generally be classified into two categories: one is to regulate its hosting gene, the other is to target different ones. We have summarized the known instances in Table 2.

### 3.1. Interfering with Parental Gene Regulation

Accumulated reports observed that circRNA can, in a complicated way, regulate its parental gene in terms of epigenetic control, splicing, transcription, or translation. For example, Chen et al. reported that *circFECR1*, a *FLI1* exonic circular RNA, interacts with the *FLI1* promoter and recruits *TET1* demethylase to induce DNA demethylation in the CpG islands. Concurrently, *circFECR1* also binds to and downregulates *DNMT1*, the critical enzyme that maintains DNA demethylation during DNA replication. Thus, *circFECR1* promotes tumor cell invasion by coordinately regulating *TET1* and *DNMT1* in breast cancers [30].

If the circRNA contains the same exon as the parental gene, the processing of circRNA will compete with the splicing of pre-mRNA. Previous studies have shown that back-splicing can compete with pre-mRNA splicing in circRNA biogenesis, resulting in low levels of linear mRNA that contain exon inclusions. In general, the more an exon is circularized, the less it presents in the processed mRNA [8,11]. 

CircRNA can also affect the transcription of its parental gene. For example, intron-retained circRNAs, such as EIciRNA are located in the nucleus. It is reported that EIciRNAs can bind to U1 snRNPs through RNA–RNA interactions, while U1 forms a complex with RNA polymerase II by binding to TFIIH. EIciRNAs regulate RNA polymerase II activity and promote parental gene transcription [28]. Similar to EIciRNA, ciRNA also can stimulate the transcription of its parental gene by up-regulating the activity of RNA polymerase II and enhance the expression of ankyrin repeat domain 52 protein (*ANKRD52*) [13]. 

Besides, some circRNAs have been demonstrated to regulate gene transcription via both RNA polymerase II complex and translation-related proteins. Recently, *circYap* was found to bind with *Yap* mRNA and the translation initiation associated proteins *eIF4G* and *PABP*. The complex containing overexpressed circYap abolishes the interaction of *PABP* on the poly (A) tail and *eIF4G* on the 5′-cap of the *Yap* mRNA, which functionally leads to the suppression of *Yap* translation initiation. Individually blocking the binding sites of *circYap* on *Yap* mRNA, or respectively mutating the binding sites for *PABP* and *eIF4G*, de-represses *Yap* translation [31]. 

### 3.2. Acting as miRNA Sponges

CircRNAs are stable in cells because they do not have a 5′end and a poly-A tail, which prevent ribonuclease degradation. Furthermore, there are multiple miRNA response elements on the circular sequences. Thus, circRNA is natural miRNA sponge. *CDR1as* consists of a single exon and 63 conserved binding sites for *miR-7*, can suppress *miR-7*’s activity, and can upregulate the expression of *miR-7* targets such as *SNCA*, *EGFR*, and *IRS2* [2]. CircRNAs can inhibit the expression of miRNAs by adsorption and, meanwhile, affect the molecular level of downstream target genes. Many studies have shown that the most general function of circRNAs is its action as a miRNA sponge to regulate target gene expression by inhibiting miRNA activity. One circRNA can regulate one or multiple miRNAs through multiple miRNA binding sites. CircRNAs regulate gene expression through binding to and releasing miRNAs from their downstream target genes. For example, we found *circTADA2A* was repressed in a large cohort of triple-negative breast cancer (TNBC) patients compared to adjacent normal tissues. Through bioinformatics analyses, *miR-203a-3p* and *miR-302c-3p* binding sites were identified in *circTADA2A*, which was confirmed in subsequent wet experiments. Functional analysis indicated *circTADA2A-E6* binds with *miR-203a-3p*, thus affecting its downstream gene SOCS3 to suppress cell migration, invasion, and clonogenicity. Furthermore, their down-regulation was correlated with survival time in TNBC patients. We conclude that *circTADA2A-E6* is a tumor-suppressor circRNA and could be utilized as a promising prognostic biomarkers and therapeutic target for TNBCs [32]. Interestingly, an oncogenic circRNA in TNBC was also discovered in a recent report. Yang et al. found that *circAGFG1* was significantly up-regulated in TNBC, and it promoted TNBC progression through the *circAGFG1/miR-195-5p/CCNE1* axis [33]. The same circRNA may even target different miRNAs to exert opposite functions in different cancers. For example, *circHIPK3* targets *miR-7* to promote colorectal cancer growth and metastasis, while it also targets *miR-558* to suppress the expression of *HPSE* and inhibits migration, invasion, and angiogenesis of bladder cancer cells in vitro [34,35].

### 3.3. Binding to Proteins

Some circRNAs that harbor binding sites for RNA-binding proteins may serve as protein sponges or decoys in addition to acting as miRNA sponges. For instance, the circular RNA *Ccnb1* could bind with *H2AX* in *p53* mutant cells and suppress mutant *p53* in tumor progression. This study found that *circ-Ccnb1* could interact with both *Ccnb1* and *Cdk1* proteins, thus counteracting the effects of *p53* mutations in breast cancer [36]. In colorectal cancer tissues, increasing *AMPK* activation is usually associated with upregulated expression of *circACC1* [37]. *CircACC1* functions to stabilize and promote the enzymatic activity of the *AMPK* holoenzyme by binding with the regulatory β and γ subunits. Argonaute 2 (*AGO2*) is a core component of the miRNA-induced silencing complex. Recently, Chen et al. identified that circAGO2, one intronic circRNA generated from the *AGO2* gene, binds with the *HuR* protein to facilitate *HuR*-repressed functions of *AGO2*–miRNA complexes in gastric cancer [38]. CircRNA can also form functional complexes with proteins. *CircFOXO3*, which is highly expressed in noncancer cells, could arrest the function of *CDK2* and block cell cycle progression via formation of a *circFOXO3–p21–CDK2* ternary complex [39]. Undoubtedly, to coordinate the different cellular processes, some of the proteins that bind with circRNA may also be an essential regulator of the biogenesis of circRNA [24].

### 3.4. Translating Proteins or Peptide

circRNAs were initially defined as a distinct class of endogenous noncoding RNA that could not translate proteins. Recently, strong evidence from several research groups has shown that circRNAs can encode proteins. Liang et al. reported that *circCTNNB1* produces a novel, 370 amino acid *CTNNB1* (i.e., *β-catenin*) isoform that uses the start codon as the linear *β-catenin* mRNA transcript, and translation is terminated at a new stop codon created by circularization. They found that this novel isoform can stabilize full-length *β-catenin* by antagonizing *GSK3β*-induced *β-catenin* phosphorylation and degradation, leading to activation of the *Wnt* pathway [40]. In addition, Zhang et al. reported that circular *lncRNA-PINT* can be translated into a small peptide to suppress glioblastoma cell proliferation; this action is mediated by trapping *PAF1c* to inhibit translational elongation of oncogenes [41]. However, the molecular mechanism of translation remains largely unknown. Generally speaking, linear mRNA translation normally requires a 5′end 7-methylguanosine (m7G) cap structure and a 3′ poly-A tail. CircRNA may be translated into peptides and proteins in other novel ways because circRNAs lack both caps and poly(A) tails. One report indicated that circRNAs can be translated from an artificial internal ribosomal entry site (IRES) to generate a functional green fluorescent protein (*GFP*) [42]. And another mechanism is that circular RNA containing an infinite open reading frame (ORF) can be efficiently translated to produce proteins [43]. Based on ribosome footprinting assays of fly head extracts, Pamudurti et al. identified that a group of circRNAs is translated in a cap-independent manner. They share the start codon with the hosting RNA, encoding specific domains from hosting protein [44].

## 4. Perspectives

In recent years, considerable interest has focused on circRNA in cancers. In this review, we outline current understandings on its biogenesis, molecular mechanism, and function, but there are also some open questions. Firstly, controls of circRNA and its parental gene are thought to be intensely coupled together [11,30,31]. However, Smid et al. developed a new circRNA prediction method, which did not rely on unmapped reads or known splice junctions. When analyzing random-primed cDNA libraries from a large primary breast cancer cohort, they found only a small part of circRNAs could be explained by known splicing events [45]. Although it cannot completely rule out the technical disparities between this study and previous investigations, it raises the possibility that specific regulatory processes and functions for circRNAs exist. Future studies are needed to systematically compare the datasets and analysis methods to clarify the relationship between circRNA and its host gene. 

Besides, genetic or epigenetic factors contributing to circRNA regulation have not been fully explored in cancers. For example, genomic aberrations include both chromosome translocation and short nucleotide variants [46]. Canonical short variants such as inversions, insertions, translocations, deletions, and duplications may introduce or interrupt elements in flanking regions; thus, they should contribute to the formation of circRNAs. Although short repeats affect circRNA production, as found in a previous reporter assay, it should be proven in clinical samples that some of the well-established cancer-associated short variants correlate with cancers via circRNA [17]. Thus, a high-throughput bioinformatics pipeline should be developed to analyze the large cancer sequencing datasets available in the public repository. In a recent analysis of sequencing data from the dorsolateral prefrontal cortex, Liu et al. combined the expression levels of circRNAs with genetic cis-acting SNPs and found that partial *circQTL* SNPs might influence circRNA formation by altering the canonical splicing site or the reverse complementary sequence match [47]. It would be interesting to conduct a similar approach in cancer samples and to see if some of these *circQTL* SNPs are highly linked to genome-wide association study signals in different cancer types. 

Finally, circRNAs possess several characteristics that are well-suitable to serve as biomarkers in cancers. Some circRNAs have tissue-specific expressions and correlate with clinical indicators [4,9,32]. They are protected from endonuclease degradation and are stable in formalin-fixed, paraffin-embedded (FFPE) tissues [48]. Notably, recent reports have shown that circRNAs can be secreted in exosomes, saliva, and urine [9,49,50]. Therefore, circRNAs could be further explored to be utilized as a surrogate for cancer diagnostics.

## Figures and Tables

**Table 1:** RNA-binding proteins that regulate circRNA biogenesis.

Gene	Effect on the Formation of circRNA	Mechanisms	PMID
*QKI*	Promote	*QKI* enhances circRNA formation by binding to its consensus target single-stranded RNA (ssRNA) motif in introns that flank circRNA-forming exons.	25768908
*TNRC6A*	Promote	The RNA-binding protein *TNRC6A* binds to the flanking intron sequence of *circHOMER1* and regulates the formation of *circHOMER1*. In the absence of *TNRC6A*, *circHOMER1* cannot be effectively looped.	29726904
*NF90/NF110*	Promote	*NF90/NF110* stimulates circRNA formation by promoting the stability of intron complementary sequences	28625552
*HNRNPL*	Promote	Circular RNA formation regulated by *HNRNPL* back splicing.	28611215
*MBL/MBNL1*	Promote	*MBL/MBNL1* strongly and specifically binds with *circMbl* flanking introns, which contain conserved muscleblind binding sites and strongly affects *circMbl* biosynthesis	25242144
*ESRP1*	Promote	The splicing factor *ESRP1*, which is controlled by the core pluripotency-associated factors, *OCT4* and *NANOG*, can lead to the promotion of circBIRC6 generation.	29074849
*FUS*	Inhibit	*FUS* was found to regulate circRNA generation by binding introns that flank back-splicing junctions	28358055
*DHX9*	Inhibit	*DHX9* can bind to IRAlus and possesses RNA helicase activity. It was speculated that *DHX9* may inhibit circRNA expression by unwinding RNA pairs that flank circularized exons.	28355180
*ADAR1*	Inhibit	*ADAR1* binds double-stranded RNA to mediate adenosine-to-inosine (A-to-I) RNA editing to inhibit circRNA formation.	25558066

**Table 2:** Different mechanism of circRNAs in human cancers.

Function	CircRNA	Cancer Type	Expression	Targeting miRNA or Genes	Mechanisms	PMID
Acting as miRNA sponge	*circAGFG1*	TNBC	Up	*miR-195-5p/CCNE1*	*CircAGFG1* can promote TNBC cell proliferation, mobility, and invasion as well as tumorigenesis and metastasis in vivo by acting as a ceRNA (competing endogenous RNA) of *miR-195-5p* to relieve the repressive effect of *miR-195-5p* on its target cyclin E1 (*CCNE1*).	30621700
	*circHIPK3*	CRC	Up	*miR-7/FAK, IGF1R, YY1, EGFR*	*circHIPK3* promotes proliferation/migration	29549306
	*circCDR1*	ESCC	Up	*miR-7/HOXB13*	*CirsCDR1* functions as the sponge of *miR-7* and reactivates its downstream *HOXB13*-mediated *NF-κB/p65* pathway.	30082829
	*circHIPK3*	BCa	Down	*miR-558/HPSE*	*circHIPK3* targets *miR-558* to suppress the expression of *HPSE* and inhibits migration, invasion, and angiogenesis of bladder cancer cells in vitro and suppresses bladder cancer growth and metastasis in vivo.	28794202
	*circTRIM33-12*	HCC	Down	*miR-191/ TET1*	*CircTRIM33-12* upregulate *TET1* expression by sponging *miR-191*, resulting in significantly reduced 5-hydroxymethylcytosine (*5hmC*) levels in HCC cells.	31153371
	*circTADA2As*	TNBC	Down	*miR-203a-3p/SOCS3*	*circTADA2As* suppresses cell proliferation, migration, invasion, and clonogenicity and possesses a tumor-suppressor capability.	30787278
	*circLARP4*	GC	Down	*miR-424-5p/ LATS1*	*circLARP4* is mainly localized in the cytoplasm and inhibits biological behaviors of GC cells by sponging *miR-424*.	28893265
	*circATP2B1*	ccRCC	Down	*miR-204-3p/FN1*	*CircATP2B1* is suppressed by *ERβ*, and then reduces *miR-204-3p*, which increases fibronectin 1 expression and enhances ccRCC cell invasion.	29490945
	*circITCH*	BCa	Down	*miR-17, miR-224/p21, PTEN*	*circITCH* promotes the aggressive biological behaviors of BCa via up-regulating the expression of *p21* and *PTEN* through ‘sponging’ *miR-17* and *miR-224*	29386015
	*circMTO1*	HCC	Down	*miR-9/ p21*	*circMTO1* can down-regulate *p21* by acting as the sponge of oncogenic *miR-9* to suppress hepatocellular carcinoma progression.	28520103
	*circDB*	HCC	Up	*miR-34a/USP7*	*CircDB* promotes tumor growth and reduces DNA damage by suppressing *miR-34a* and activating the *USP7/Cyclin A2* signaling pathway	30546088
Binding to proteins	*CircACC1*	CRC	Up	*AMPK*	*CircACC1* functions to stabilize and promote the enzymatic activity of the *AMPK* holoenzyme by forming a ternary complex with the regulatory β and γ subunits.	31155494
	*circDNMT1*	BRCA	Up	*P53, AUF1*	Ectopically expressed *circDnmt1* promotes the nuclear translocation of both *p53* and *AUF1*, p53 nuclear translocation induces cellular autophagy, while *AUF1* nuclear translocation reduces *Dnmt1* mRNA instability, resulting in increased *Dnmt1* translation.	29973691
	*circAGO2*	GC	Up	*HuR*	*circAGO2* binds with *HuR* protein to promote its activation and enrichment on the 3′-untranslated region of target genes, which reduces *AGO2* binding and repression of *AGO2*/miRNA-mediated gene silencing	30341421
	*circPABPN1*	Hela cell	Up	*HuR*	The binding of *CircPABPN1* to *HuR* inhibits *HuR* binding to *PABPN1* mRNA and reduces *PABPN1* translation.	28080204
	*circFOXO3*	BRCA	Down	*circFOXO3, p53*	*CircFoxo3* promotes *MDM2*-induced *p53* ubiquitination and subsequent degradation by binding to *Foxo3* protein and *p53*, resulting in cell apoptosis.	27886165
	*circCCNB1*	BRCA	Down	*Ccnb1/Cdk1*	*CircCcnb1* can interact with both *Ccnb1* and *Cdk1* proteins. Ectopic delivery of *circCcnb1* inhibits tumor growth and extends mouse viability.	31199987
Translating proteins or peptide	*circCTNNB1*	HCC	Up	370-amino acid β-catenin isoform	*CircCTNNB1* produces a novel, 370 amino acid *β-catenin* isoform that uses the start codon as the linear *β-catenin* mRNA transcript, and translation is terminated at a new stop codon created by circularization.	31027518
	*CircPINT*	GBM	Up	*PINT87*aa	*lncRNA-PINT* can be translated into a small peptide to suppress glioblastoma cell proliferation	30367041
	*CircE7*	Derived from human papillomavirus and presented in CESC and HNSC	Up	*E7* protein	Specific disruption of *circE7* in CaSki cervical carcinoma cells reduces *E7* protein levels and inhibits cancer cell growth both in vitro and in tumor xenografts.	31127091
	*circSHPRH*	GBM	Down	*SHPRH*-146aa	*SHPRH*-146aa is a tumor suppressor in human glioblastoma, which is translated by *circ-SHPRH*.	29343848
	*circFBXW7*	GBM	Down	*FBXW7*-185aa	The spanning junction open reading frame in *circ-FBXW7* driven by internal ribosome entry site encodes a novel 21 kDa protein (*FBXW7*-185aa). Upregulation of *FBXW7*-185aa in cancer cells inhibits proliferation and cell cycle acceleration.	28903484
Regulating parental gene expression at multiple levels	*circFECR1*	BRCA	Up	*TET1, DNMT1.*	*CircFECR1* regulates DNA methylation and demethylation enzymes to control breast cancer tumor growth.	30537986
	*circYAP*	BRCA	Up	*eIF4G, PABP*	*CircYap* can bind with *Yap* mRNA and the translation initiation associated proteins, *eIF4G* and *PABP*, which functionally leads to the suppression of *Yap* translation initiation.	31092884
	*circEIF3J, circPAIP2*	Hela, HEK293	Up	*U1 snRNA*	EIciRNAs predominantly localizes in the nucleus, interacts with U1 snRNP, and promotes transcription of their parental genes.	25664725

BRCA: breast cancer; GC: Gastric cancer; BCa: bladder cancer; CRC: colorectal cancer; CESC: cervical squamous cell carcinoma and endocervical adenocarcinoma; HNSC: head and neck squamous cell carcinoma; GBM: primary glioblastomas; HCC: hepatocellular carcinoma; TNBC: Triple-negative breast cancer; ccRCC: Clear Cell Renal Cell Carcinoma; and ESCC: esophageal squamous cell carcinoma.

## Abbreviations

circRNA	Circular RNA
lncRNA	Long noncoding RNA
ciRNAs	Circular intronic RNAs
EIciRNAs	Exonic-intronic circRNAs
TricRNA	tRNA intronic circular RNA
RBPs	RNA-binding proteins
siRNA	Small interfering RNA
hESCs	Human embryonic stem cells
TCGA	The Cancer Genome Atlas
PMID	PubMed Unique Identifier
SNPs	Single nucleotide polymorphisms
